# Anesthetic Management for Multiple Family Members with Myotonic Dystrophy for Interventional Cardiac Procedures—A Case Series

**DOI:** 10.3389/fmed.2017.00243

**Published:** 2018-01-08

**Authors:** Leonid Gorelik, Antolin Flores

**Affiliations:** ^1^Anesthesiology, The Ohio State University Wexner Medical Center, Columbus, OH, United States

**Keywords:** myotonic muscular dystrophy presentation, anesthetic care of patients with MMD, general anesthesia vs. monitored anesthesia care for patients with myotonic dystrophy, aspiration risk with myotonic dystrophy, pacemaker insertion in patients with myotonic dystrophy

## Abstract

Myotonic muscular dystrophy (MMD) is a rare autosomal dominant disorder that can complicate anesthetic management of patients. MMD is characterized by progressively worsening muscle loss and weakness, cardiac conduction abnormalities, cardiomyopathy, restrictive lung disease, obstructive sleep apnea, and delayed gastric emptying. Patients presenting with MMD for any surgical procedure present a management challenge to the anesthesiologist. Several reports of airway loss due to medication-mediated respiratory depression, sudden death due to dysrhythmias, aspiration of stomach contents, and prolonged intubation have been reported. We present a case series of three family members with MMD type 1 who presented for electrophysiologic assessment of the cardiac conduction system and possible pacemaker insertion. While there are reports of anesthetic management of patients with myotonic dystrophy for various procedures, our report is unique in that we were able to demonstrate variations of anesthetic management based on the procedure and variation in disease phenotype—differing severity between family members.

## Introduction

Myotonic muscular dystrophy (MMD) is a rare autosomal dominant disorder that can complicate anesthetic management of patients. It is the most common form of adult-onset muscular dystrophy (1). MMD is characterized by progressively worsening muscle loss and weakness. It affects the skeletal muscle and the smooth muscle of the body (2), leading to prolonged muscle contractions and impaired relaxation. Other symptoms include cataract formation, cardiac conduction abnormalities, cardiomyopathy, restrictive lung disease, obstructive sleep apnea, delayed gastric emptying, infertility, and, in severe cases, cognitive dysfunction (3). There are two most common types of MMD, type 1 and type 2. They differ in their genetic mutations as well as manifestations. MMD type 1 is caused by mutations in the DMPK gene, whereas type 2 is caused by mutations in the CNBP gene (1). The muscular weakness present in MMD type 1 usually affects the face, neck, hands, and lower legs, while the weakness associated with MMD type 2 usually affects the neck, shoulder, elbows, and hips (1).

Patients presenting with MMD for any surgical procedure present a management challenge to the anesthesiologist. There have been several reports of airway loss due to medication mediated respiratory depression, sudden death due to dysrhythmias, aspiration of stomach contents, and prolonged intubation. We present a case series of three family members with MMD type 1 who presented for electrophysiologic (EP) assessment of the cardiac conduction system and possible pacemaker insertion. While there are reports of anesthetic management of patients with myotonic dystrophy for various procedures, our report is unique in that we were able to demonstrate variations of anesthetic management based on the procedure and variation in disease phenotype—differing severity between family members.

## Methods

Institutional Review Board (IRB) exemption status granted under Ohio State Wexner Medical Center IRB. Procedures and anesthetic care for individual cases took place in November, 2016 under the direct care of the authors.

## Results/Case Series

Three siblings with known history of MMD type 1 were scheduled to undergo EP studies with possible device placement under anesthesia over a span of 3 weeks. Per family report, their mother, who also had MMD type 1, died from aspiration pneumonia after an endoscopy 3 years prior. As a result, the family was very anxious about undergoing this procedure. After consultation with the family, the decision was made to try to avoid the use of neuromuscular blockade.

The first patient was a 31-year-old female, presenting for an EP study with possible pacemaker placement. The patient was able to ambulate without the use of a walker or braces. She reported the ability to walk a considerable distance before becoming fatigued. As a part of the MMD disease process, the patient was also diagnosed with central sleep apnea and used a CPAP machine at night. She denied any history of gastroesophageal reflux disease (GERD). The decision was made to proceed with the EP study under monitored anesthesia care (MAC) and convert to general anesthesia (GA) if a pacemaker needed to be placed. The patient received Ondansetron 4 mg IV, Metoclopramide 10 mg IV, and Dexamethasone 4 mg IV at the start of the procedure in order to prevent nausea, vomiting, and possible aspiration. For sedation, she was given Fentanyl 50 mcg IV, Midazolam 2 mg IV, and was started on a Propofol infusion. She tolerated the study well.

When the EP study revealed a prolonged PR interval (217 ms) and a wide QRS (125 ms), the cardiology team decided to proceed with pacemaker placement. At this time, the decision was made to convert to GA and secure the airway. The patient was given an additional bolus of Propofol (80 mg IV) and Fentanyl (50 mcg IV) and was successfully intubated using direct laryngoscopy without the use of paralytic agents. She tolerated this well and was maintained on a Propofol infusion for the duration of the procedure. On emergence from GA, the patient received Ondansetron 4 mg IV and was extubated uneventfully after she was taking adequate tidal volumes, protecting her airway, and following commands. She was discharged home the next day with no acute events.

The second patient was a 25-year-old male, the younger brother of the first patient. He presented for an EP study with possible pacemaker placement. The patient reported generalized weakness and poorer functional status than his sister, necessitating the use of assistance to ambulate. However, in contrast with his sister, this patient denied a history of sleep apnea or the use of a CPAP machine (polysomnogram was reportedly negative). He also denied any history of GERD. This patient had a similar anesthetic plan prescribed, using MAC anesthesia for an EP study and converting to GA if a pacemaker needed to be placed. For sedation, the patient received a total of 100 mcg of Fentanyl IV (in 50 mcg boluses) and 2 mg of Midazolam IV (in 1 mg boluses). He required minimal sedation for the study and was comfortable throughout. His EP study did not reveal any ECG abnormalities (PR interval 200 ms, QRS 110 ms), and there was no need for pacemaker insertion at the time. The patient emerged from sedation without incident and recovered in the post-anesthesia care unit without evidence of respiratory compromise. He was discharged home the same day with recommendations for serial ECGs every 6–12 months.

The third patient, the middle sibling of the first two patients, was a 27-year-old male presenting for an EP study with possible pacemaker placement. The patient reported generalized weakness as well but was able to ambulate without any assistance. He had a history of central sleep apnea and used a CPAP machine at night, similar to his sister, the first patient. On physical exam, it was noted that the patient had extremely poor dentition with multiple erythematous areas, suspicious for infection and/or abscess. After discussion with the electrophysiology team, it was agreed that if the patient needed pacemaker insertion, he would need to obtain dental clearance prior to placement due to high risk of device infection. However, the EP study could still proceed. The plan was to use MAC anesthesia for the study and have the patient return for a subsequent anesthetic if a pacemaker was indicated. He received Dexamethasone 4 mg IV, Famotidine 20 mg IV, Metoclopramide 10 mg IV, and Ondansetron 4 mg IV for nausea and vomiting prophylaxis. For the procedure, the patient received a total of 100 mcg of Fentanyl IV (in 50 mcg boluses) and 2 mg of Midazolam IV (in 1 mg boluses). Interestingly, during the study, the patient developed atrial fibrillation with rapid ventricular response, and heart rate as high as the 150 s. His blood pressure was stable throughout the event. The decision was made to cardiovert the patient. Propofol 30 mg IV bolus was given, and cardioversion was performed without the need for intubation. The patient subsequently returned to normal sinus rhythm. The EP study showed abnormal infranodal conduction and pacemaker placement was recommended. However, due to poor dentition and concern for tooth abscess, pacemaker placement was deferred. After satisfactory recovery from sedation and no evidence of respiratory compromise or apneic events, the patient was discharged home.

## Discussion

This case series documents the anesthetic care of three siblings with MMD type 1. Each sibling had different degrees of disease manifestation and severity. The first and third patients both had generalized weakness but were able to ambulate without the assistance of a walker. The second patient, however, did require assistance with ambulation. In addition, the first and third patients were also diagnosed with central sleep apnea and used a CPAP machine at night. All of these factors contributed to their respective anesthetic management.

The anesthetic plan for all three siblings was to start with MAC sedation and convert to GA if a device was indicated. Only the first of the three patients required conversion to GA for pacemaker insertion. While the third patient would have benefited from device placement as well, his extremely poor dentition at the time of the procedure increased the risk of infecting a newly placed device significantly. The decision point between MAC and GA is a very important one for these patients, especially those with more severe disease burden. A retrospective study by Mathieu et al. of 219 patients demonstrated an odds ratio of 14.1 for postoperative respiratory complications in MMD patients with proximal limb weakness undergoing GA versus MMD patients without (4). While GA has been reportedly administered safely in MMD patients with more benign disease, many common agents administered during a routine GA pose significant concerns—opioids, neuromuscular blockers (5). Thus, the risks and benefit assessment needs to be well communicated with the patient, anesthesia team, and the surgical or procedural team.

Monitored anesthesia care sedation has the advantage of avoiding intubation and maintaining protective airway reflexes; however, many procedures cannot be safely accomplished using this technique. Another complicating factor is that sedation for these patients has to be very minimal. If they are even mildly over-sedated without a secure airway, there is a significant risk of airway loss or aspiration due to lower baseline pharyngeal muscle tone (3).

In contrast, GA provides a secure airway for the procedure. However, these patients are at an increased risk of aspiration on induction of GA. In addition, there is an increased risk for prolonged intubation, which can lead to other complications such as ventilator-associated pneumonia, deconditioning, and chronic respiratory failure requiring a tracheostomy. As demonstrated by the first case, the conversion to GA was successfully accomplished with the use of propofol and fentanyl but no paralytic agents, in order to minimize any effects on the patient’s musculoskeletal system. Neuromuscular blocking agents (paralytics), depolarizing and non-depolarizing, may have altered effects on this patient population, but case reports to date have been inconsistent. Succinylcholine has been reported to cause dose-dependent contractures in MMD patients and should be avoided (3). While non-depolarizing agents are generally well-tolerated, the antagonizing agent, neostigmine, has been reported to incompletely reverse neuromuscular blockade (3) and even cause a myotonic response (6). However, with the recent approval of sugammadex, safe and successful reversal of rocuronium has been reported with this patient population (7).

All three patients were very closely monitored for arrhythmias throughout the procedure. The third patient developed atrial fibrillation with rapid ventricular response during the study and had to undergo cardioversion. The other two patients did not develop any arrhythmias.

In summary, taking care of patients with myotonic dystrophy can pose a significant challenge to the anesthesiologist in the operating room but may be safely conducted with the proper considerations. Patients with this condition are at risk for aspiration, airway loss, prolonged intubation, and cardiac arrhythmias. Careful consideration must be given to the type of anesthetic required for the procedure. A careful and thorough risk and benefit assessment must be communicated with the patient as well as the surgical team. If MAC is chosen, it is imperative to avoid over-sedating the patient. If GA is chosen, it is preferable to avoid the use of paralytic agents. However, if muscle relaxation is required for the given procedure, then full and adequate reversal of the paralytic agent is required with demonstration of adequate muscular function prior to extubation. Additional monitoring for muscle weakness in the PACU or ICU can be beneficial as well.

## Ethics Statement

This case series was IRB exempt. Written informed consent was obtained.

## Author Contributions

LG and AF took care of two of the patients described in this case series. Both authors wrote and edited the manuscript.

## Conflict of Interest Statement

The authors declare that the research was conducted in the absence of any commercial or financial relationships that could be construed as a potential conflict of interest.
